# Overdiagnosis of mental disorders in children and adolescents (in developed countries)

**DOI:** 10.1186/s13034-016-0140-5

**Published:** 2017-01-17

**Authors:** Eva Charlotte Merten, Jan Christopher Cwik, Jürgen Margraf, Silvia Schneider

**Affiliations:** 1 Department of Clinical Child and Adolescent Psychology of the Faculty of Psychology, Ruhr-Universität Bochum, Massenbergstraße 9-13, 44787 Bochum, Germany; 2 Department of Clinical Psychology and Psychotherapy of the Faculty of Psychology, Ruhr-Universität Bochum, Massenbergstraße 9-13, 44787 Bochum, Germany

**Keywords:** Overdiagnosis, Child and adolescent psychiatry, Mental disorders, ADHD, Heuristics

## Abstract

During the past 50 years, health insurance providers and national registers of mental health regularly report significant increases in the number of mental disorder diagnoses in children and adolescents. However, epidemiological studies show mixed effects of time trends of prevalence of mental disorders. Overdiagnosis in clinical practice rather than an actual increase is assumed to be the cause for this situation. We conducted a systematic literature search on the topic of overdiagnosis of mental disorders in children and adolescents. Most reviewed studies suggest that misdiagnosis does occur; however, only one study was able to examine overdiagnosis in child and adolescent mental disorders from a methodological point-of-view. This study found significant evidence of overdiagnosis of attention-deficit/hyperactivity disorder. In the second part of this paper, we summarize findings concerning diagnostician, informant and child/adolescent characteristics, as well as factors concerning diagnostic criteria and the health care system that can lead to mistakes in the routine diagnostic process resulting in misdiagnoses. These include the use of heuristics instead of data-based decisions by diagnosticians, misleading information by caregivers, ambiguity in symptom description relating to classification systems, as well as constraints in most health systems to assign a diagnosis in order to approve and reimburse treatment. To avoid misdiagnosis, standardized procedures as well as continued education of diagnosticians working with children and adolescents suffering from a mental disorder are needed.

## Background

During the past 50 years, a worldwide increase in prevalence rates of mental disorders in children and adolescents was found in studies using data from health insurance providers [1], national registers of health services [2, 3], and special education programs [4]. Furthermore, studies using data from national registers of drug prescriptions found that prescription rates of psychoactive medication have increased [5]. Regarding attention-deficit/hyperactivity disorder (ADHD), the rate of psychostimulant use in children and adolescents in some studies exceeds earlier prevalence rates of ADHD (8–10% of students in grades 2 through 5 in two cities received medication for ADHD) [6]. Research shows that children who do not fulfill ADHD criteria are treated with psychostimulants [7]. These findings have raised concerns regarding overdiagnosis of ADHD in daily practice, especially as a recent study reported prevalence rates up to 20% [1], much too high to attain by definition of the disorder as a cluster of age-inappropriate behavior. Reviews using epidemiological data examining time trends in prevalence rates of mental disorders in children and adolescents have shown mixed results. One review found an increase in prevalence of autism over time [8], while others showed differing results, depending on the disorder explored [9], or no increase in prevalence at all [10–12]. It needs to be noted, that two of these reviews [9, 12] do not report how the diagnoses were established and another review [10] included studies defining cases based on questionnaire scores or “judgment by interviewee”. Therefore, on one hand, we do not know if the reported time trends of prevalences of mental disorders in the general population truly reflect only the cases fulfilling diagnostic criteria for mental disorders. On the other hand, we know that the number of children and adolescents diagnosed with and treated for mental disorders has skyrocketed over the past decades. At the same time, underdiagnosis and undertreatment represent serious problems. The World Health Report published 2001 by the World Health Organization [13] showed that many countries lack sufficient mental health resources and sometimes mental health policy altogether. Although underdiagnosis represents a serious problem, with children and adolescents not getting the help they need, this paper focuses on the overdiagnosis of mental disorders.

Various explanations, as well as their combinations, might be responsible for this phenomenon: (1) Growing awareness of mental disorders and an accompanying reduction in stigmatization could lead to greater health care utilization. Children and adolescents, who remained underdiagnosed in the past, might receive a correct diagnosis and treatment today. (2) Improved diagnostic procedures may have led to better identification of mental disorders. (3) Changes in diagnostic criteria lead to reduced thresholds for a diagnosis, resulting in increases in prevalence rates following each published version of the Diagnostic and Statistical Manual of Mental Disorders (DSM) for ADHD [14–16] and autism spectrum disorder (ASD) [8]. (4) Diagnosticians may not strictly adhere to diagnostic criteria. Instead, their clinical judgment is affected by heuristics and biases.

Examining the hypothesis of overdiagnosis in mental disorders reveals a diagnostic dilemma unique to mental disorders. Unlike somatic disorders, mental disorders cannot be detected by genetic, neuronal, or physiological correlates. Rather, they compose of a research-supported consensus of expert-defined clusters of feelings and behaviors described in diagnostic manuals like DSM or the International Classification of Diseases (ICD). Hence, research concerning diagnostic accuracy is based on research on reliability, since mental disorders lack external criteria for examining validity.

Therefore, it is difficult to examine the hypothesis of overdiagnosis as an explanation for the increase in prevalence rates. As stated above, it remains uncertain whether a given diagnosis is “true”. It can only be examined if the diagnostician adhered strictly to the diagnostic criteria. We, therefore, define overdiagnosis as assignment of a diagnosis, although diagnostic criteria were not met. Furthermore, *false*-*positive* cases must occur more often than *false*-*negative* cases, where a diagnosis is not given although diagnostic criteria are fulfilled [17].

Research concerning overdiagnosis or factors that influence diagnosis in children and adolescents is sparse, while some disorders are more researched than others. Overdiagnosis and overmedication in ADHD receives broad attention and is widely researched; most studies found in our literature search dealt with ADHD. Some investigations also focused on bipolar disorder (BD), ASD, psychotic disorders, anxiety disorders, learning disorders, and mental disorders in children and adolescents in general.

In the present paper, we address (1) the topic of overdiagnosis by conducting a systematic literature search and reporting evidence for or against overdiagnosis and (2) summarize research concerning factors that might cause misdiagnoses in child and adolescent mental disorders.

### Evidence for overdiagnosis of mental disorders in children and adolescents

We conducted a systematic search of literature using Medline, PsychINFO, PubMed, and Web of Science in April 2014, with the following keywords: child, youth, adolescent, psychology, psychiatry, overdiagnosis, false-positive, misdiagnosis.

Studies were eligible for inclusion if they: (1) included children or adolescents; (2) investigated mental disorders; (3) presented the results of peer-reviewed research; (4) and examined diagnostic accuracy, for example, via re-evaluation of diagnoses or diagnostic agreement.

**Fig. 1:**
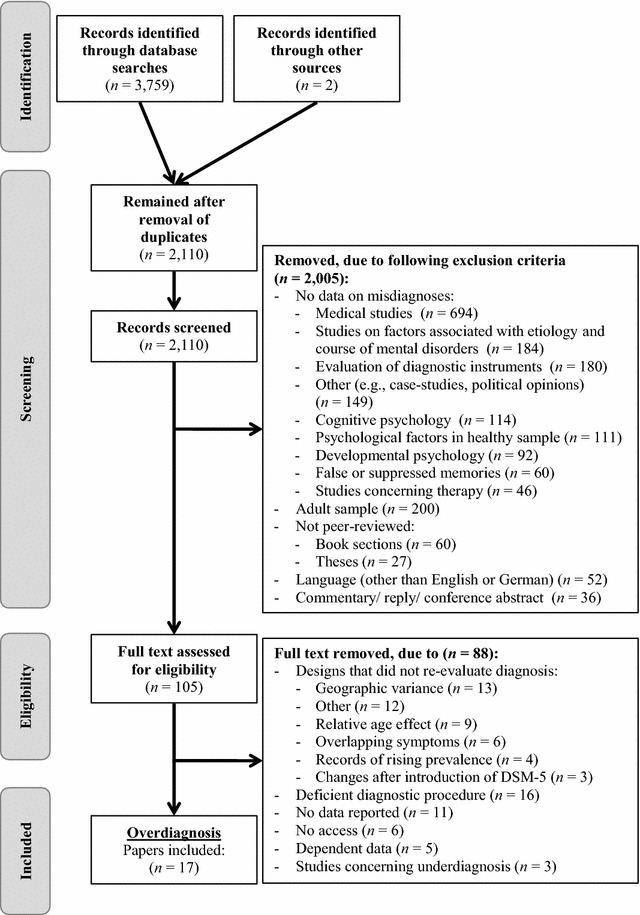
Flow diagram of study selection procedure

Case studies, theses and dissertations, papers not published in peer-reviewed journals, and trials published in languages other than English or German, as well as papers examining false-positives in questionnaires used for screening purposes or studies concerning questionnaire validations were excluded. A multi-step selection strategy was used (see Fig. 1). First, duplicate studies were excluded. Then, titles and abstracts of all studies were screened for inclusion and exclusion criteria. When we were in doubt whether a study would meet the inclusion criteria, it was included in the next stage.

For the second part of this article, selected studies of high quality or reviews were chosen from the previously excluded papers. Thus, while the first part is a systematic review, the second part of the paper presents a non-systematic overview.

Studies found in the literature search varied in their capacity to confirm overdiagnosis. Table 1 shows the main characteristics of the studies and main results with respect to overdiagnosis. To examine the hypothesis of overdiagnosis, the first group of studies (see Table 1) re-evaluated diagnoses, either by evaluating earlier diagnosis or by following the long-term stability of diagnoses that are by definition profound and should not change dramatically, like autism. These studies compared the diagnoses of psychiatric inpatients [18–23], diagnoses made at intake to outpatient clinics [24, 25] or diagnoses made by mental health professionals [26–31] with diagnoses based on a strict application of diagnostic criteria for example by the use of a clinical (semi-)structured interview. Studies concerning mental disorders in general in children and adolescents [22, 24, 25, 28] found very low agreement for individual diagnoses between clinician-generated and interview-generated diagnoses, respectively, for inpatient and subsequent outpatient diagnoses [21] or between pre-admission diagnoses and diagnoses made in a specialized diagnostic and treatment center for patients with developmental disabilities [30]. In the study by Jensen and Weisz [25], reevaluation resulted in a higher number of diagnoses than formerly assigned by clinicians. This seems to speak against the hypothesis of overdiagnosis in every-day clinical routine. Two other studies reported higher prevalence of mood disorder diagnoses in inpatient-diagnoses, although re-evaluation via clinical interview [28], respectively, subsequent outpatient-diagnoses [21] showed a higher prevalence of ADHD and disruptive behavior disorders. All other studies dealt with the reevaluation of particular disorders like ADHD [26, 27], BD [18, 20], psychotic disorders [19, 23, 29] or agoraphobia [32] in children and adolescents. In these studies, a substantial number of children and adolescents lost their former practitioner-generated diagnoses after reevaluation. Wiggins et al. [31] analyzed data on the stability of ASD diagnoses. They found that only 4% changed to non-ASD diagnoses. In contrast, Woolfenden et al. [33] reviewed 23 studies examining the stability of diagnoses of autism. While 85–88% kept their diagnosis of ASD, stability for Asperger syndrome or ASD (not otherwise specified) was significantly lower with 14–61% keeping their diagnosis unchanged at follow-up.

**Table 1:** Studies evaluating overdiagnosis

Author(s) (year)	Diagnosis	N	Study	Result
First group of studies: re-evaluating former diagnoses				
Chilakamarri and Filkowski (2011) [26]	ADHD, BD, MDD (DSM-IV)	n = 64 patients (age 7–18)	Re-evaluation of former diagnoses at intake in a community primary care mental health setting	Overdiagnosis of ADHD, underdiagnosis of BD, over- and underdiagnosis of MDD
Cotugno (1993) [27]	ADHD (DSM-III-R)	n = 92 patients (age 5–14)	Re-evaluation of former diagnoses at intake in a specialized ADHD clinic	22% of former ADHD-cases were given a primary diagnosis of ADHD, 37% a secondary diagnosis of ADHD
Ezpeleta et al. (1997) [24]	DSM-III-R diagnoses	n = 137 patients (age 6–17)	Agreement between clinician-generated diagnoses at intake in an outpatient clinic and diagnoses given after a clinical interview	Low to moderate agreement
Jensen and Weisz (2002) [25]	DSM-III-R diagnoses	n = 245 patients (age 7–17)	Agreement between clinician-generated diagnoses at intake in an outpatient clinic and diagnoses given after a clinical interview	Low agreement, more diagnoses after clinical interview
Krasa and Tolbert (1994) [18]	BD (DSM-III-R)	n = 53 patients (age 13–18)	Re-evaluation of diagnoses after discharge from an inpatient psychiatric service	28.3% received an other diagnosis after re-evaluation (MDD, organic mood disorder, schizophreniform disorder, posttraumatic stress disorder, conduct disorder, ADHD, developmental receptive language disorder)
Lewczyk et al. (2003) [28]	DSM-IV diagnostic categories	n = 240 patients (age 6–18)	Agreement between discharge diagnoses generated by county mental health providers and diagnoses given after a clinical interview	Low overall agreement; higher prevalence of ADHD, disruptive behavior disorder and anxiety disorders based on clinical interview; higher prevalence of mood disorders based on clinical diagnoses
McClellan et al. (1993) [19]	Psychotic disorders (DSM-III-R)	n = 39 patients (age 7–17)	Re-evaluation of diagnoses given at an inpatient psychiatric clinic after m = 3.9 years	Diagnoses changed at follow up: 46% of schizophrenia, 66% mood disorder, 40% personality disorder
McKenna et al. (1994) [29]	Schizophrenia (DSM-III-R)	n = 71 patients (age 8–18)	Re-evaluation of diagnoses given at major academic centers	73% received a diagnosis other than schizophrenia after evaluation
Pogge et al. (2001) [20]	BD (DSM-IV)	n = 29 patients (age mean 15.18)	Agreement between clinical chart diagnoses at psychiatric inpatient clinic and research-quality assessment, involving structured interviews	40% of clinical chart diagnoses confirmed by research-quality assessment 79% of research-quality diagnoses confirmed by clinical-chart diagnoses
Safer (1995) [21]	DSM-III-R diagnoses	n = 82 youth patients	Comparison between inpatient and subsequent outpatient diagnoses	Low agreement, inpatient: mostly mood-disorder diagnosis, outpatient: mostly disruptive behavior disorders
Sevin et al. (2003) [30]	DSM-IV diagnoses	n = 150 adolescents (age 11–19) with developmental disabilities	Comparison between pre-admission diagnoses and diagnoses made in a dual diagnosis treatment unit, serving adolescents with a developmental disability and a mental disorder	Less externalizing, psychotic and mood disorders after re-evaluation, more Tic and substance related disorders
Vitiello et al. (1990) [22]	DSM-III diagnoses	n = 46 patients (age 6–13)	Agreement between chart diagnoses in a child psychiatry inpatient unit, diagnoses given after structured clinical interviews with the child and the patient’s parents and review diagnoses given after discharge by reviewing all relevant information regarding the child’s psychopathology	Disagreement between chart and structured interview diagnoses in 1/3 of cases Agreement between review-diagnoses and chart diagnoses: 67% Structured interview (parents): 87% Structured interview (children): 69%
Werry et al. (1991) [23]	Psychotic disorders (DSM-III-R)	n = 61 patients (age 7–17)	Re-evaluation of former diagnosis after m = 5 years	55% of bipolar diagnoses at follow up had a former diagnosis of schizophrenia
Wiggins et al. (2012) [31]	ASD (DSM-IV-TR)	n = 1392 child patients	Analysis of data from education and health records in surveillance years 2000 and 2006	4% changed in classification to non-ASD (mostly to language delay or disorder or other specific developmental delay)
Wittchen et al. (1998) [32]	Agoraphobia (DSM-IV)	n = 173 patients (age 14–24)	Re-evaluation of structured interview diagnosis by clinical psychologists	Agoraphobia diagnosis was confirmed in 13.9% of cases; mostly patients received specific phobia diagnoses after re-evaluation
Woolfenden et al. (2012) [33]	ASD (DSM-III–DSM-IV-TR, ICD-9, ICD-10)	n = 1466 child patients	Review of 23 studies concerning stability of ASD diagnoses	Moving out of the ASD spectrum at follow up with a former autistic disorder diagnosis (other ASD) Baseline age <3 years: 0–30% (0–53%) Baseline age 3–5 years: 0–20% (0–5%) Baseline age >5 years: 0–16% (0–23%)
Second group of studies: designs able to prove overdiagnosis				
Bruchmüller et al. (2012) [34]	ADHD (DSM-IV, ICD-10)	n = 463 German child and adolescent psychotherapists	Evaluating case-vignettes fulfilling/not fulfilling criteria of ADHD	16.7% diagnosed ADHD, although criteria were not fulfilled vs. 7.0% not diagnosed with ADHD although criteria were fulfilled

*ADHD* attention-deficit/hyperactivity disorder, *ASD* autism spectrum disorder, *BD* bipolar disorder, *MDD* major depressive disorder

At first glance, these studies seem to confirm overdiagnosis, as diagnoses were changed after re-evaluation, indicating that diagnoses were given although criteria were not met. However, it remains unclear if there were more false-positive than false-negative diagnoses, therefore, there is no clear proof for overdiagnosis. Further it remains unclear at which point in the diagnostic process the errors took place. It could be that diagnosticians assigning the initial diagnoses lacked important information. Just as well, diagnosticians might have had all relevant information, but made false interpretations. However, if the diagnostic decisions of raters who are provided with all relevant information for a diagnosis are compared, possible mistakes could be traced back to the decision-making process and explicit proof of overdiagnosis would so be provided. Our literature search found only one study using such a study design (see Table 1).

Bruchmüller et al. [34] sent case vignettes describing a child fulfilling or not fulfilling diagnostic criteria for ADHD to 473 child and adolescent psychotherapists and asked them to indicate which diagnosis they would assign. In total, eight case vignettes differing by diagnostic status and gender of the child were used. In total 16.7% of psychotherapists diagnosed ADHD although diagnostic criteria were not fulfilled. Only 7% gave no diagnosis, although the case vignette fulfilled diagnostic criteria for ADHD. Therefore, there were significantly more false-positive than false-negative diagnoses, which can be seen as proof of overdiagnosis of ADHD in this study.

Further, ADHD was diagnosed two times more often in the boy-version of the case vignettes, reflecting a common finding in ADHD research that more males are diagnosed with ADHD than females. Similar to findings concerning the time trends in prevalence of mental disorders mentioned above, there is a difference between clinical data, with male to female ratios between 5:1 and 9:1, and epidemiological data with ratios of approximately 3:1 [35]. The differences in symptom expression of this disorder between boys and girls could lead to an easier detection of boys with ADHD [35]. Bruchmüller et al. [34] assumed further, that the diagnostic decision of raters is influenced by representativeness heuristics. That is, as more boys than girls are affected by ADHD, boys with ADHD-like symptoms are seen as more similar to prototypical ADHD cases. Therefore, diagnosticians may neglect the base rate of ADHD and the correct application of diagnostic criteria in favor of a so-called rule of thumb.

The use of heuristics in the diagnostic process is one possible explanation for the observed differences between clinical and epidemiological data in mental disorders. Further, these studies show that diagnosticians are prone to making mistakes in the decision-making process. While the literature search detected only few studies specifically examining overdiagnosis, we identified a number of studies which suggest that misdiagnosis does occur. Due to their respective study designs, these studies cannot contribute to the question whether more false positive than false negative diagnoses occur and therefore cannot shed light on the question of overdiagnosis. However, by identifying factors influencing the diagnostic process, they can indicate how to reach more reliable diagnostics. In the second part of this article, we summarize this topic by referring to reviews or selected original studies of high quality.

### Factors that might cause misdiagnoses in child and adolescent mental disorders

Factors that influence diagnosis can be assigned to two steps of the diagnostic process. First, information concerning the behavior and feelings of a patient need to be assessed. Different to mental disorders in adults, mental disorders in children are established using a multi-informant approach. Thus, not only the child but also the parents and other important caregivers (e.g., teachers) are asked for a description of the child's behavior. Second, the diagnostician must decide whether the gathered information point to a diagnosis. The process of information gathering is prone to mistakes due to factors concerning the informant. The diagnostic decision-making process can be influenced by multiple factors, for instance by the characteristics of the diagnostician, the diagnostic criteria or the health care system in question.

### Information gathering

#### Influence of factors concerning the informant

In their assessment of information, diagnosticians depend on the description of symptoms by the respective informant. Like diagnosticians, also informants are prone to heuristics, illustrated by two studies asking teachers to describe children’s behavior. Teachers viewed videotapes of child actors engaging in normal behavior, behavior typically seen in ADHD or oppositional defiant disorder [36, 37]. Teacher ratings of hyperactivity were higher for child actors who showed oppositional behavior than for those showing ‘normal’ behavior. Independent raters rated the two videotapes equally concerning hyperactivity, pointing to a halo effect. The halo effect is a cognitive bias where factors that seem important for a decision influence all other information taken into consideration in the decision-making process. Further, Jackson and King [37] found that hyperactivity ratings for a male child actor showing oppositional behavior were significantly higher than ratings for a female child actor. This demonstrates the tendency to overrate male externalizing behavior, which was confirmed by Bruchmüller et al. [34].

Parents as informants may also be vulnerable to biases and the use of heuristics. Weckerly et al. [38] found that caregivers with higher levels of education tend to endorse more inattention-symptoms of ADHD, while endorsement of hyperactivity-symptoms was shown to be unrelated to the educational level of the informant. Further, maternal psychopathology in some studies was found to be associated with higher ratings of psychopathology by mothers in their children, compared to teacher ratings [39], ratings of healthy counterparts, and self-report of the 14-year-old offspring [40].

Additionally, some studies found that children and adolescents with externalizing disorders can show a so-called positive illusory bias (PIB) [41]. That is, they rate themselves as significantly more positive than their parents, teacher or other raters. PIB has been associated with less effective social behavior [41] and with less benefit from treatment [42]. However, on the positive side, participants with PIB reported fewer depressive symptoms [42]. Nevertheless, biases in self-evaluation in connection with other mental disorders and their consequences for diagnostics and treatment need further attention in research.

Concluding, the use of heuristics and biases in judgment of child and adolescent behavior not only apply to diagnosticians, but to their informants as well. As diagnosticians cannot fully rely on informants’ judgment of the child’s behavior, it is crucial to take multiple sources of information into account, including self-reports of the children and adolescents as even the discrepancy between evaluations might give substantial hints for treatment planning. Studies show that even very young children with externalizing psychopathology, who were formerly considered to be unreliable informants [43], can provide valuable information concerning their symptomatology if an age-appropriate approach is used [44].

#### Influence of factors concerning characteristics of the child or adolescent

Children and adolescents may express symptoms of mental disorders differently from adults. For example, DSM-5 diagnostic criteria of major depression disorder state that children might not show sad, but irritable mood [45]. Depressed children might report unspecific somatic complaints [46] or depression might result in attention problems, leading to misdiagnosis of depressed children as having learning disorders [47]. Similarly, adolescents with substance abuse might show symptoms of learning disabilities [48].

A large body of ADHD research shows that children born close to kindergarten or school cut-off dates, and who are therefore young compared to their classmates, are between 30 and 60% more likely to be diagnosed with ADHD [3, 49] and receive psychostimulants twice as often as children born only a few days later, but after the cut-off date [3, 49, 50]. Elder [49] found this effect in US states with different cut-off dates, pointing to a relative age effect, rather than to a season of birth effect assumed by earlier studies. Translated to the American population, this means that “approximately 1.1 million children received an inappropriate diagnosis [of ADHD] and over 800,000 received stimulant medication due only to relative [im]maturity” [51]. The relative age effect was found not only in the United States [49, 51], but also in Canada [3], Sweden [52], and Iceland [50] and was shown to be stable over an 11-year period [3].

Goodman et al. [53] examined the relative age effect for all mental disorders, in a sample of 10,438 children between 5 and 15 years in England, Scotland, and Wales. They found an increase in risk of psychopathology with decreasing relative age in all three countries. This also points to a relative age effect rather than to a season of birth effect, as the three countries have different cut-off dates.

This finding could partly explain the overdiagnosis of ADHD and other disorders too; diagnosticians misinterpret children’s developmentally normal behavior as symptoms of a mental disorder by considering merely children’s numeric age, rather than their age in relation to the age of their peers.

In summary, it is vital that diagnosticians assessing children or adolescents are well trained in child development and symptom-expression in various age groups.

### Decision-making

#### Influence of factors concerning the diagnostician

As a reason for overdiagnosis, especially in the male version of the case-vignettes, Bruchmüller et al. [34], assume that the diagnostician’s clinical judgment concerning ADHD is affected by heuristics. Rather than adhering strictly to diagnostic criteria, diagnosticians may base their judgments on principal similarities [54] or weigh the criteria differently. Studies on learning disorders [55], mania [20, 56], and agoraphobia [57] in children and adolescents also found that diagnosticians give more weight to criteria that seem more predominant for a certain diagnosis or overlook exclusion criteria which might be considered insignificant.

Besides the use of heuristics to determine if criteria are fulfilled, diagnosticians also interpret behavior as fulfilling criteria differently. After reviewing case vignettes of ADHD [58] or prepubertal mania [59], the diagnoses of researchers and clinicians in the US and the UK differed according to their nationality, indicating a representative heuristic due to national diagnostic practice. Furthermore, the application of DSM or ICD, which are designed for flawless diagnoses of mental disorders by operationalizing each disorder in diagnostic criteria, showed low reliability in an international context. This indicates that diagnostic criteria are not operationalized sufficiently to guarantee flawless recognition of a disorder.

#### Influence of factors concerning diagnostic criteria

Another factor possibly hindering a correct diagnosis is the overlapping of symptoms of two mental disorders. Three symptoms overlap between ADHD and BD. Considering the high comorbidity between these two disorders [60], an overdiagnosis due to overlapping symptoms is distinctly possible.

Milberger et al. [61] reevaluated cases with ADHD and comorbid BD diagnoses by subtracting shared symptoms. Additionally, they adjusted the required symptoms for a diagnosis to match the original criteria. Discarding overlapping symptoms resulted in a rejection of BD diagnosis in more than half of the cases in this sample. ADHD diagnosis remained even after the exclusion of overlapping BD symptoms. This points to an overdiagnosis of BD due to common symptoms with ADHD, since an ADHD diagnosis is not an exclusion criterion for BD.

In regard to exclusion criteria, the diagnostic criteria of ADHD also contain risks, since they lack an exclusion criterion due to medical conditions. Inclusion of such a criterion would be important, as studies show that medical conditions like sleep apnea can result in symptoms that resemble ADHD but will disappear if the medical condition is resolved [62]. These studies emphasize the importance of interpreting symptoms in the context of other disorders in order to correctly diagnose mental disorders.

Changes in the diagnostic systems DSM and ICD are another important factor concerning diagnostic criteria influencing diagnostics. For example, in DSM-5, Asperger’s disorder was integrated into the broader category social communication disorder and the threshold for age of onset for ADHD was lowered. Such changes may present difficulties in research, as diagnoses now include patients with possibly different characteristics or formerly subdivided groups of patients are now under the same diagnosis. More importantly from the patient perspective, this might lead to problems regarding access to service and treatment [63].

#### Influence of factors concerning the health systems

Literature also suggests intentional overdiagnosis due to health policy constraints.

As in many health care systems a diagnosis is required in order to access and reimburse treatment, intentional wrong coding in diagnosing mental disorders does occur in child and adolescent mental health services and can partly account for the overdiagnosis found in studies reevaluating earlier diagnoses. Clinicians might intend to ensure help for children with unclear or borderline symptoms or want to proceed with an evaluation without denying treatment when it is too early to render a diagnosis.

Because a diagnosis is required for the approval and reimbursement of interventions and treatment, clinicians in the study of Jensen and Weisz [25] were significantly more likely to assign just one diagnosis and significantly less likely to refrain from diagnoses for their inpatients compared to the results based on a structured interview. More distinct evidence was found in two studies using questionnaire surveys with pediatricians and child psychiatrists exploring the frequency and possible reasons for wrong coding. In the first study [64], 58% of participants reported that in order to provide their patients with educational ascertainment support, they had given an ASD diagnosis although they were not sure if the diagnosis was appropriate. Only four participants reported doing so although they knew for certain that the child did not have ASD. In the second study [65], 2/3 of the participants reported intentional wrong coding due to diagnostic uncertainty, inadequate diagnostic criteria, or economic issues.

## Implications for daily practice and further research

Although rarely researched, first indications of overdiagnosis of child and adolescent mental disorders are evident. Especially the study of Bruchmüller et al. [34] provides strong evidence for overdiagnosis in ADHD. To qualify the results, the generalization of the study must be questioned, as only German psychotherapists were included. Further, the ecological validity is questionable, as diagnosing case vignettes may lack the feeling of responsibility of a real diagnostic situation, also not allowing therapists to further inquire about diagnostically relevant behaviors. On the other hand, using case vignettes which clearly state or exclude certain diagnostic criteria should have facilitated the decision making process as case vignettes control for variance in the process of data gathering.

However, the evidence base is too weak to draw definite conclusions about the extent of overdiagnosis in children and adolescents. To assess the degree of overdiagnosis in daily practice, more research with study designs that contrast false-positive with false-negative diagnoses is needed. Nevertheless, research points to different factors that may lead to mistakes in the diagnostic process, providing starting points for the improvement of diagnostic quality. The most important factor seems to be low interrater reliability for mental disorders in everyday clinical routine, due to heuristics and insufficient application of diagnostic criteria.

One study showed that only 1/4 of pediatricians report relying on DSM criteria [66] although diagnostics based on established criteria is associated with more accurate diagnoses than decisions based on professional judgment [55]. Hence, in order to reduce misdiagnosis due to insufficient use of diagnostic criteria, one could argue based on these results that the use of clinical interviews as the gold-standard in diagnosing mental disorders [67] should be more clearly promoted in the training of pediatricians, if the respective health care systems allow pediatricians to diagnose and treat mental disorders. In some countries, only mental health specialists are allowed to treat and diagnose mental disorders. Dalsgaard et al. [68] found no relative age effect in a sample of 416,744 Danish children. Their conclusion was that the risk of diagnosing children of relative young age is lower if only specialists are allowed to diagnose ADHD, as is the case in Denmark. The study by Abikoff et al. [36] also points to the importance of expertise in gathering information for diagnostic decisions, as the halo effect in teacher ratings of hyperactivity was found only in regular, not in special education teachers. Still, research showed that also experts like child and adolescent psychotherapists and psychiatrists overdiagnose ADHD [34]. Nevertheless, most studies suggest that expertise at least reduces the risk of diagnostic mistakes in dealing with externalizing disorders. Therefore, special and continuing education for those diagnosing mental disorders in children and adolescence is needed.

Health policy regulations can substantially impact diagnostic quality since they can assure that only trained practitioners using standardized procedures can diagnose mental disorders in order to reduce the risk of misdiagnoses. Further, health policy has a substantial impact on treatment options, as is shown in two studies exploring the influence of prescription monitoring [69] and drug insurance programs [70] on the magnitude of psychostimulant use. Hence, future studies should compare the effect of different health care systems internationally and explore the effects of changes in these systems in order to identify characteristics that might contribute to better diagnoses and lead to more valid and careful handling of mental disorders. In an ideal world, health policy should enable practitioners to diagnose a certain disorder unaffected from financial or political aspects, ensuring each person in need access to service and treatment.

Additionally, diagnostic criteria in standardized assessment procedures themselves are partly imprecise. The relative age effect reveals that children born just before the cut-off date for schooling can fulfill the diagnostic criteria for ADHD and would seem to benefit from medication, although their behavior might be part of a normal course of neurodevelopment taking place in a different environment compared to their same-age peers, who remain in kindergarten a year longer. Beside this evidence for low validity of diagnostic criteria, at least in the case of ADHD, it is evident that diagnostic criteria are not reliable enough, as even trained clinicians interpret same symptoms differently [58].

Consequentially, new ways for the classification of mental disorders are currently under consideration. The research domain criteria framework introduced by the NIMH [71] attempts to classify mental disorders as disorders of brain circuits, including data from clinical neuroscience to the clinical symptoms. The cognitive behavior model by Hofmann [72] rejects the idea of mental disorders as specific latent disease entities. Instead it “classifies mental disorders using a complex casual network perspective” [72]. Thus, both frameworks avoid classification problems due to misinterpretation of observed behavior that meets the criteria of different disorders.

## Conclusion

While there is little research concerning overdiagnosis of child and adolescent mental disorders, first studies point to misdiagnosis of several mental disorders. Unintended overdiagnosis can occur due to use of heuristics, disregarding differential causes of observed behavior, misleading endorsement of symptoms by caregivers, or differential interpretation of diagnostic criteria by examiners.

To resolve this problem and to ascertain that children and adolescents are not harmed by unnecessary (medication-) treatment, clinicians diagnosing mental disorders are encouraged to use semi-structured clinical interviews and should actively participate in continuous education regarding latest findings in research, while diagnostic criteria must undergo constant evaluation in order to meet the latest state of scientific knowledge.

## Abbreviations

ADHD: attention-deficit/hyperactivity disorder
ASD: autism spectrum disorder
BD: bipolar disorder
DSM: Diagnostic and Statistical Manual of Mental Disorders
ICD: International Classification of Diseases
PIB: positive illusory bias
